# Exposing the latent phenotype of Gulf War Illness: examination of the mechanistic mediators of cognitive dysfunction

**DOI:** 10.3389/fimmu.2024.1403574

**Published:** 2024-06-11

**Authors:** Hannah E. Burzynski, Lawrence P. Reagan

**Affiliations:** ^1^ Department of Pharmacology, Physiology, and Neuroscience, University of South Carolina School of Medicine, Columbia, SC, United States; ^2^ Department of Psychology, Binghamton University, Binghamton, NY, United States; ^3^ Columbia Veterans Affairs (VA) Health Care System, Columbia, SC, United States

**Keywords:** acetylcholinesterase, pyridostigmine bromide, acetylcholine, hippocampus, lipopolysaccharide, stress

## Abstract

Though it has been over 30 years since the 1990–1991 Gulf War (GW), the pathophysiology of Gulf War Illness (GWI), the complex, progressive illness affecting approximately 30% of GW Veterans, has not been fully characterized. While the symptomology of GWI is broad, many symptoms can be attributed to immune and endocrine dysfunction as these critical responses appear to be dysregulated in many GWI patients. Since such dysregulation emerges in response to immune threats or stressful situations, it is unsurprising that clinical studies suggest that GWI may present with a latent phenotype. This is most often observed in studies that include an exercise challenge during which many GWI patients experience an exacerbation of symptoms. Unfortunately, very few preclinical studies include such physiological stressors when assessing their experimental models of GWI, which creates variable results that hinder the elucidation of the mechanisms mediating GWI. Thus, the purpose of this review is to highlight the clinical and preclinical findings that investigate the inflammatory component of GWI and support the concept that GWI may be characterized as having a latent phenotype. We will mainly focus on studies assessing the progressive cognitive impairments associated with GWI and emphasize the need for physiological stressors in future work to create a more unified hypothesis that can identify potential therapeutics for this patient population.

## 1 Introduction

Nearly one third of soldiers that served in the 1990–1991 Gulf War (GW) continue to suffer from an unexplained, multi-symptom illness (1). While the potential for a “Gulf War Illness” (GWI) was initially debated by the Departments of Defense (DoD) and Veterans Affairs (VA), it was ultimately recognized as a unique combination of symptoms solely affecting GW Veterans in 1997 (2, 3). This recognition came after multiple case studies and congressional hearings reported significantly higher rates of unexplained health issues and poorer overall health in GW Veterans compared to other cohorts of Veterans from this era (4, 5). Such symptoms included cardiovascular abnormalities such as tachycardia and hypertension, gastrointestinal (GI) disturbances including diarrhea and irritable bowel syndrome (IBS), and respiratory impairments including emphysema and chronic bronchitis (1, 6). Many GW Veterans also reported neurological disturbances including chronic headaches, sleep disturbances, persistent depression, and memory and attentional impairments (1, 7).

As the symptomology of GWI is quite varied, the state of Kansas created the Kansas Persian Gulf War Veterans Health Initiative Program to establish explicit criteria for GWI diagnoses (8). This work identified six main categories of GWI symptomology: fatigue/sleep, somatic pain, neurologic/cognitive/mood, GI symptoms, respiratory symptoms, and skin symptoms (8). Now known as the Kansas definition, GWI is diagnosed if a patient reports symptoms in at least three of these six categories (8). The Center for Disease Control established a broader definition of GWI that only includes mood/cognition, fatigue, and musculoskeletal categories (9). GWI is diagnosed using these criteria if a patient reports one chronic symptom in two of the three categories (9). Though different, both the Kansas and CDC definitions have been recommended for diagnostic use by the DoD and VA.

Utilizing these standardized definitions, it is estimated that GWI affects approximately 200,000–250,000 of the 700,000 troops deployed to the GW (1). While GWI has been reported in both men and women, the majority of cases are seen in men as over 95% of GW Veterans are male (6). GWI is not only complex in its symptomology, but also in its progression as 10-year and 20-year follow-up surveys reported that many GWI symptoms had worsened in affected Veterans (7, 10, 11). This progression includes declines in neurological measures, including cognitive function and neuropsychiatric disorders (7, 12, 13), which coincides with volume loss in the cerebellum and brainstem of some patients (14, 15). For additional information on the initiation and progression of GWI, readers are referred to Gulf War and Health Volumes 1–11 from the Institute of Medicine Committee on Gulf War and Health. Adding to its complexity, many symptoms of GWI, including cognitive deficits, are exacerbated when patients experience stressors and many GW Veterans complain of severe post-exertional malaise (16). Such reports suggest that GWI may present with a latent phenotype, as the full symptomology may only appear after a stressor is presented. Unfortunately, the number of preclinical and clinical studies examining these stimulus-dependent symptoms are limited. Thus, the purpose of this review is to illustrate the latent phenotype of GWI and to highlight the importance of including physiological stressors in future GWI studies in order to fully elucidate the mechanisms driving this complex pathology, as well as to identify potential therapeutics.

## 2 GWI and inflammation

While this complex disease remains poorly understood, immune dysfunction is now considered a hallmark feature of GWI as numerous clinical studies have found a persistent pro-inflammatory state in GWI patients. Early studies reported that relative to controls, GWI patients exhibited elevated numbers of plasma T cells and B cells, while the number of natural killer cells was reduced approximately 2–8 years after their GW service (17, 18). Additionally, studies found higher plasma levels of pro-inflammatory markers such as interferon-gamma (IFN-γ), interleukin (IL)-2, and tumor necrosis factor alpha (TNF-α) in GWI patients at similar post-deployment time points (17, 19). This immune profile has been validated by more recent studies that also found elevations in these cytokines, as well as IL-6 and C-reactive protein (CRP) in samples collected between 2010 and 2013 (20, 21). Along with higher levels of pro-inflammatory cytokines, another study found a down regulation of the anti-inflammatory cytokines IL-4 and IL-13 in the serum of GWI patients almost 25 years after their GW service (22). Chronic inflammation resulting from sustained elevations of pro-inflammatory cytokines in plasma has been implicated in various diseases such as IBS, rheumatoid arthritis, and psoriasis, all of which have been reported in patients with GWI (6, 7, 12, 23–25). Recently, similarities between GWI and post-acute COVID-19 syndrome (PACS) or “long COVID” symptomology have been drawn, suggesting that this chronic inflammation may be due to an unidentified, persistent antigen (26). Thus, while the mechanistic basis of this immune dysfunction has not been fully elucidated, it is likely that the aberrant immune signaling is contributing to a broad range of pathological settings, including GWI.

Beyond its peripheral effects, systemic inflammation is also known to produce neuroinflammation through humoral, cellular, and neural pathways (27). This includes the passage of circulating immune cells and cytokines into the central nervous system (CNS) through various transport mechanisms (28). Additionally, vagal afferent fibers innervating the periphery can detect the presence of pro-inflammatory signals and relay this information to the CNS (29). Collectively, this detection of peripheral inflammation triggers an inflammatory response within the CNS that can compromise the overall health and function of neurons when such pathways are chronically activated. As such, some researchers have hypothesized that chronic, peripheral inflammation may be a mechanistic mediator in the cognitive decline observed in GWI patients through the initiation of chronic neuroinflammation. For example, the cytokine high-mobility group box 1 (HMGB1) is elevated in plasma samples of GWI patients (30) and intraperitoneally (i.p.) administered HMGB1 has been shown to activate microglia and produce memory impairments and depressive-like behavior in mice (31–34). Similar conclusions were drawn in a separate study that found that adding serum of GWI patients to neural cultures caused a reduction in cell growth and an increase in apoptosis which disrupted neural network communication (35). These investigators suggested that such responses occurred because the serum of GWI patients contains neuropathogenic factors as these impairments were ameliorated by the addition of serum from healthy GW Veterans (35). This neuroinflammatory component of GWI is further supported by recent findings that visualized greater amounts of activated microglia and astrocytes in the brains of GWI patients relative to healthy GW Veterans and civilians with *in vivo* imaging (36). Taken together, these studies identify neuroinflammation as a putative causative factor in the neurological complications in GWI patients, but additional studies are needed to identify specific CNS biomarkers and fully elucidate the mechanism(s) responsible, including the contribution of peripheral immune dysfunction.

Along with immune dysfunction, dysregulated endocrine signaling has also been implicated in GWI (6, 7, 16). These impairments have been associated with abnormal hypothalamic-pituitary-adrenal (HPA) axis activity, which regulates the stress response by producing cortisol through the release of corticotropin releasing factor (CRF) and adrenocorticotrophin hormone (ACTH) (37). HPA axis dysfunction in GWI subjects was observed following a dexamethasone suppression test, which assesses the feedback activity of the HPA axis by mimicking cortisol release which should suppress ACTH and in turn, the release of cortisol (38). Specifically, approximately 15 years after the GW, GWI patients exhibited a greater suppression of cortisol levels than controls, which may be indicative of impaired pituitary or adrenal responses (39). Golier and colleagues expanded upon these findings and reported that GW Veterans exhibit significantly lower levels of plasma ACTH and a higher cortisol:ACTH ratio, which they attributed to an enhanced sensitivity to cortisol’s feedback effects and reduced HPA axis activity (40). Importantly, cortisol and other glucocorticoids released by the HPA axis perform critical anti-inflammatory actions and the attenuation of this axis in GW Veterans may be exacerbating the observed immune dysfunction (41, 42).

## 3 Potential causes and mechanisms of GWI

In view of these clinical observations, peripheral and/or central inflammation is proposed to be a mechanistic mediator of GWI pathology. As such, it is important to identify potential exposures during deployment that could be responsible for these inflammatory responses in GWI Veterans. Soldiers were exposed to several hazards in the Gulf including the use of pesticides and insecticides, airborne particulates from dust and sand, depleted uranium in military vehicles, smoke from oil well fires, and severe stress from combat (43). Additionally, soldiers were administered various vaccines and pyridostigmine bromide (PB), a pharmacological prophylaxis against potential nerve gas attacks (44, 45). While the list of harmful exposures during the GW is extensive, it is interesting that multiple exposures, namely PB, nerve gases, pesticides/insecticides, and stress, all greatly impact the cholinergic system, a key regulator of cognition and anti-inflammatory responses in both the periphery and CNS.

### 3.1 Pyridostigmine bromide and nerve gases

PB is the dimethylcarbamate ester of 3-hydroxy-1methylpyridinium bromide that was originally used for the treatment of myasthenia gravis (MG) as it acts an acetylcholinesterase (AChE) inhibitor (46, 47). PB blocks the enzymatic activity of AChE by binding to the serine residue in the enzyme active site, rendering it inactive (48). Being a carbamate compound, PB is considered a reversible AChE inhibitor as the binding of the AChE-carbamate complex that inhibits AChE action is quite unstable and will spontaneously split from the enzyme by hydrolysis (49). Importantly, PB’s inhibitory actions are confined to the periphery due to its quaternary ammonium group that limits lipid solubility and prevents passage through the blood-brain barrier (BBB) (50). While PB is well known for its therapeutic properties, many other AChE inhibitors are better known for their deleterious actions. Organophosphate (OP) AChE inhibitors, such as some nerve gases, are much more toxic than PB as they permanently inhibit the enzyme by non-reversible phosphorylation (49). Such irreversible AChE inhibitors produce a dangerous accumulation of acetylcholine (ACh) in the synaptic cleft that compromises normal muscle control, which becomes lethal if the respiratory muscles are affected and asphyxiation occurs (51). OP AChE inhibitors have been used as chemical warfare agents as they can be vaporized or aerosolized to attack large groups through their inhalation and absorption into the skin (48).

Saddam Hussein was thought to possess large stockpiles of these OP AChE inhibitors during the GW, namely the G-series nerve agents, sarin and soman (52). PB was identified as a potential prophylactic against irreversible OP AChEs due to its ability to compete for AChE binding (53). Preclinical studies before the GW showed that carbamate AChEs, such as PB, significantly improved survival rates after nerve agent exposure when used as a pretreatment (44, 45, 53). Thus, PB was used as an investigational drug in the GW, with each soldier receiving 21-tablet blister packs and instructed to take one 30 mg tablet every 8 hours to ensure continuous protection (46, 54). This prescribed dose suppressed plasma AChE activity by approximately 50% (55), which replicated the dose tested in preclinical models (45). Many soldiers reported immediate side effects to PB, with most involving the GI system, but such symptoms were not considered incapacitating (56). The rationale for this prophylactic approach had practical implications, especially in view of reports from the DoD estimating that perhaps 100,000 personnel were possibly exposed to low levels of sarin nerve agent after the Khamisiyah munitions depot demolition (57). More recent studies have correlated higher rates of mild cognitive impairment (MCI) and smaller hippocampal volumes in GW Veterans that recall hearing chemical alarm sounds more frequently, which was attributed to greater nerve agent exposure, but the prophylactic use of PB during these incidents is not clear (58). Indeed, while the DoD reports that approximately 250,000 military personnel took PB during the GW, its self-administration led to a wide variety of dosing regimens (59). Despite its varied use, PB exposure has been consistently correlated with GWI presentation as many GWI symptoms can be attributed to cholinergic toxicity (43, 60–62). Currently, the use of PB for MG is not attributed to any long-term side effects beyond mild GI disturbances (63), but adverse effects have not been widely studied in individuals without MG, or when combined with other ACh-altering exposures.

### 3.2 Pesticides/insecticides

Another form of prophylaxis during the GW was the liberal use of pesticides and insecticides as a means to control vector-borne diseases. Due to this aggressive approach, the DoD has estimated at least 41,000 servicemembers may have been overexposed to such chemicals, including chlorpyrifos, permethrin, and *N,N*,-diethyl-meta-toluamide (DEET). Importantly, like PB, these chemicals are known to alter AChE activity (50, 64, 65). Specifically, chlorpyrifos is an irreversible, OP AChE inhibitor similar to nerve gases that acts as an extremely potent pesticide (66). Chlorpyrifos and other OP AChE inhibitors were used during the GW as fumigating agents for campgrounds (67). Permethrin is a synthetic pyrethroid and insecticide that causes respiratory paralysis in arthropods by blocking sodium channels (65). This chemical was applied to soldiers’ uniforms and skin through aerosol sprays (68). While it is not classified as an AChE inhibitor, permethrin has been shown to inhibit AChE in multiple brain regions when administered to rats intragastrically (65). Lastly, DEET is a common insect repellent used in the GW that works through similar mechanisms as permethrin and is thought to have weak AChE activity (69). As with PB, the use of such pesticides/insecticides during the GW varied greatly among soldiers, but their neurotoxic effects have been proposed as a causative factor of GWI.

### 3.3 War-related stress

Though many of the aforementioned GW exposures have been linked to GWI, the incidence of GWI is more prevalent in ground forces that faced combat compared to forces that remained in relief areas (8). This disparity led researchers to hypothesize that the physical and psychological stress of combat may also contribute to GWI presentation (70–76). As in most military conflicts, GW soldiers experienced several psychological stressors, including the lack of social contact with family, threat of attack, and fear of a friend or self being killed (77). Importantly, though such stressors are known to produce post-traumatic stress disorder (PTSD) in Veteran populations, studies have concluded that the symptomology of GW Veterans is distinct from PTSD (78). While PTSD has been shown to exacerbate GWI symptoms (78), adjusting for PTSD diagnoses revealed worse overall health outcomes in GW Veterans relative to non-GW Veterans from this era, independent of PTSD prevalence (79).

As described above, the stress response is primarily mediated by the HPA axis and chronic stress, such as the prolonged stress of deployment, can dysregulate the feedback mechanisms of the HPA axis and lead to systemic inflammation, and both are seen in GWI patients (19, 37). Along with peripheral inflammation, chronic stress can affect the central nervous system (CNS) by disrupting the brain regions that regulate the stress response. This includes the hippocampus, which is an essential integration center for learning and memory in the mammalian brain (80). From a morphological perspective, chronic stress reduces neurogenesis of dentate gyrus granule neurons and induces dendritic atrophy of pyramidal neurons within this region, which can produce memory and attentional deficits like those observed in GWI (81, 82). Similar effects of chronic stress on neuronal structure and function are also observed in the prefrontal cortex (PFC) (83), a region that mediates higher-order cognitive functions such as attention and decision making (84). While the stress response within the CNS involves numerous neurotransmitters, ACh is a key mediator as cholinergic neurotransmission increases in both the hippocampus and PFC in response to a stressor (85). This increased efflux is thought to be adaptive for acute stressors as it can promote synaptic plasticity, but more severe or chronic stress can elicit excessive ACh efflux in these brain regions that produces maladaptive stress responses and impairs cognitive function (86). Thus, the synergistic effects of AChE inhibitors and war-related stress on cholinergic neurotransmission may be especially relevant to the cognitive deficits observed in GWI.

### 3.4 Acetylcholine in GWI

#### 3.4.1 Role of acetylcholine in cognition

A large portion of the cholinergic neurons in the CNS are found in the basal forebrain (BF) nuclei, a heterogeneous set of structures located in the rostroventral forebrain (87). These structures include the medial septum and the diagonal band of Broca which send cholinergic projections to the hippocampus (88). Clinical studies have shown the importance of ACh neurotransmission in the hippocampus by using cholinergic antagonists to impair performance in memory tasks (89, 90). In addition to hippocampal projections, the BF also contains the nucleus basalis and substantia innominata, which house cholinergic projections that innervate the PFC (88). It is well established that BF cholinergic neurons are involved in PFC-dependent cognitive processes, as lesioning BF projections in rodents results in significant impairments in attentional tasks (91–95). Besides cholinergic neurons, ACh receptors are also found on other neurons in the PFC and hippocampus including glutamatergic, GABAergic, noradrenergic, and dopaminergic neurons (85). Thus, ACh is also considered a neuromodulator as it can mediate the release of these other neurotransmitters and regulate neuronal excitability, synaptic plasticity, and coordinate the firing of groups of neurons to adapt to environmental stimuli (96–99).

#### 3.4.2 The cholinergic anti-inflammatory pathway

Among its modulatory actions, cholinergic neurotransmission has also been recognized as a key regulator of inflammation through the cholinergic anti-inflammatory pathway (100). The peripheral cholinergic anti-inflammatory pathway was first identified by Kevin Tracey’s group who discovered the presence of α7 nicotinic acetylcholine receptors (nAChRs) on macrophages. (101). *In vitro* studies found that activation of α7 nAChRs by ACh reduced the expression of pro-inflammatory cytokines, specifically IL-1β, IL-6, IL-18, and TNF-α in macrophages exposed to the endotoxin lipopolysaccharide (LPS) (101). These *in vitro* studies were later confirmed *in vivo* when α7 nAChR deficient mice exhibited suppressed anti-inflammatory responses to an LPS challenge compared to wild type mice (102). Later studies identified α7 nAChRs on other immune cells including monocytes (103, 104), T-cells (105, 106), and B-cells (107).

Peripherally, ACh’s anti-inflammatory actions are mediated by the vagus nerve (100). Vagal afferent fibers innervate a multitude of peripheral organs and alert the CNS about any homeostatic disturbances through their projections to the nucleus tractus solitarius (NTS) in the brainstem (29). Vagal afferents can detect such disruptions as they contain receptors for endotoxins and the pro-inflammatory cytokines they produce (108). Once this information is relayed to the NTS, it is propagated to other brain regions involved in processing visceral information, such as the hypothalamus, amygdala, cortex, and locus coeruleus (109–111). To restore homeostasis, vagal efferent fibers residing in the dorsal motor nucleus of the vagus within the brainstem trigger the release of norepinephrine (NE) from the splenic nerve in the celiac plexus (112). NE then triggers the release of ACh from lymphocytes through β2-adrenergic receptors and ACh attenuates the release of pro-inflammatory mediators by binding to α7 nAChRs on immune cells (112). This anti-inflammatory vagal reflex is best illustrated in vagotomized mice experiencing endotoxemia after high dose LPS administration while sham animals do not (101). Importantly, the immunosuppressive actions of the vagus nerve are incredibly efficient and occur much faster than other immune responses (113).

As α7 nAChRs are also expressed in the CNS, more recent studies have investigated if a central cholinergic anti-inflammatory pathway exists. Evidence for such a pathway was first supported by studies that identified α7 nAChRs on microglia and astrocytes, key CNS immune cells (114, 115). *In vitro* studies have used ACh and nicotine, a nAChR agonist, in microglial cultures to show that activation of α7 nAChRs suppresses LPS-induced increases in TNF-α and this response is blocked by the α7 nAChR antagonist α-bungarotoxin (115). The immunosuppressive role of microglial α7 nAChRs has also been observed *in vivo* as intracerebroventricular administration of the α7 nAChRs agonist PNU282987 attenuated LPS-induced microglial activation and sickness behavior in mice (116). Astrocytic α7 nAChRs elicit similar effects as nicotine also dampens the pro-inflammatory response of cultured astrocytes activated by IL-1β (117). As neuroinflammation has been implicated in a variety of neurodegenerative diseases including Alzheimer’s disease, Parkinson’s disease, and multiple sclerosis, disruptions in the cholinergic anti-inflammatory pathway may be a mechanistic mediator of these diseases (118, 119). Indeed, many preclinical studies have investigated α7 nAChR agonists as potential therapeutics for neurodegenerative diseases driven by neuroinflammation (120–122). The therapeutic effects of α7 nAChR agonists have also been investigated in chronic inflammatory diseases in the periphery including rheumatoid arthritis, ulcerative colitis, and diabetes (123–125). This area of research may be especially relevant for GWI studies as GW Veterans experience similar inflammation-mediated symptoms and immune dysregulation (19, 126). Furthermore, many GWI symptoms involve organ systems that are highly innervated by the vagus nerve (i.e., heart, lungs, GI tract) (7, 127). Unfortunately, little is known about the potential dysfunction of the peripheral and/or central cholinergic anti-inflammatory network in GWI, including how these deficits may contribute to its latent phenotype.

### 3.5 Latent phenotype of GWI

Perhaps the best illustration of the latent phenotype of GWI emerges when patients are subjected to an exercise challenge. It is well established that fatigue and post-exertional malaise are common symptoms of GWI (16), but exercise has also been shown to exacerbate other GWI symptoms including musculoskeletal pain and cognitive impairments (128–130). Various imaging studies have been performed to understand this latent phenotype of GWI and why exercise can worsen these symptoms. One group reported that while performing a working memory task after bicycling, the dorsal midbrain and cerebella dermis became deactivated in GWI patients, but not healthy controls (129). Additionally, this bicycling challenge separated GWI patients into two phenotypes, with one group exhibiting orthostatic tachycardia and brainstem atrophy, and the other group experiencing severe pain and exhibiting cortical atrophy (131). Another study that analyzed cerebrospinal fluid after bicycling found elevations in glutamate in a subset of GWI patients, which may be indicative of exercise-induced excitotoxicity (132).

It has been suggested that such negative responses to exercise may be due to sensitized immune and endocrine responses as GWI patients subjected to a bicycle test also showed abnormal immune responses. Specifically, exercise induced a heightened pro-inflammatory state in GWI patients relative to healthy controls as the inflammatory activities of nuclear factor kappa B (NF-κB) and IL-6 were increased. Other studies from this group report that exercise reduced the expression of genes associated with the activity of natural killer cells in GWI patients (133). Interestingly, many of these genes are also involved in glucocorticoid receptor signaling pathways and salivary cortisol was significantly lower in GWI patients after exercise (133). These reductions provide further evidence that diminished HPA axis activity may be a key feature of GWI and its latent phenotype as most symptoms are exacerbated following an exercise challenge. It is important to note that these findings occurred when an aerobic, cycling challenge was used, but lower intensity exercises, such as yoga, have been shown to decrease GWI symptom severity, especially chronic pain (134, 135). Thus, the pathophysiology of GWI may be due to sensitized responses to certain stressors, but the mechanisms responsible for these aberrant immune and endocrine responses have not been fully elucidated.

## 4 Lessons from GWI studies in rodents: focus on inflammation

As noted above, clinical studies suggest that while no specific neurotoxic chemical exposure explains the illnesses observed in GW Veterans (2), administration of AChE inhibitors in combination with the stress of deployment likely contributes to the initiation and progression of GWI. In view of these observations, many preclinical studies have investigated the neurological effects of PB administration alone and in combination with pesticides and/or stress. [For a recent comprehensive review of GWI models, please see (66)]. Interestingly, though the treatment paradigms vary, inflammation is a common feature among many GWI animal models, which is consistent with clinical findings in GWI patients. For example, plasma levels of pro-inflammatory cytokines, including IL-1β, TNF-α, and IFN-γ, were significantly elevated in a rat model of GWI approximately 6 weeks after exposure to GW chemicals (PB + permethrin + DEET) and restraint stress (RS) (136). These animals also exhibited muscle atrophy, which recapitulates the musculoskeletal disturbances seen in GWI patients (136). Another group investigated the long-term effects of their GWI model (PB + permethrin + DEET + RS) and saw similar increases in plasma IL-1β and TNF-α levels, as well as IL-1α and fibroblast growth factor 2 (FGF2) levels more than 6 months after treatment (137). The same group also observed increased expression of oxidative stress markers in the plasma of these rats and reproduced these findings in a second study (137, 138).

Given the established relationship between neuroinflammation and cognitive decline, many studies have assessed neuroinflammation in rodent models of GWI, especially at delayed timepoints, as a potential cause of the progressive cognitive deficits observed in patients. Similar to plasma cytokines, elevations in pro-inflammatory cytokines (IL-1β, TNF-α, IFN-γ), were reported in the brains of a GWI mouse model (PB + permethrin) almost 1 year after the cessation of treatment (139). More region-specific studies found elevations in pro-inflammatory mediators in the hippocampus (137), and cortex (140) of a GWI rat model 6–10 months after GW chemical exposure (PB + permethrin + DEET + RS). As astrocytes and microglia are the immune cells within the CNS that drive the release of pro-inflammatory signals, many studies report an upregulation of markers for astrocyte and microglia activation in the hippocampus and cortex of GWI rodents (139, 141–143). Furthermore, some GWI rodent models exhibit decreases in neurogenesis in the dentate gyrus, as well as reduced hippocampal volume approximately 4 months after GW chemical exposure (PB + permethrin + DEET + RS) (141, 143). A more recent study reported that GWI mice (PB + chlorpyrifos + DEET) also exhibit dendritic atrophy and decreases in spine density in dentate gyrus granule neurons (144). Taken together, these studies suggest that GW chemicals produce long-lasting inflammation within the CNS which likely involves reactive astrocytes and microglia producing pro-inflammatory cytokines, thereby leading to deficits in neuronal structure. Given that these changes are occurring in the cortex and hippocampus, such findings identify a role for neuroinflammation in the progressive cognitive decline observed in many GW Veterans suffering from GWI.

While these studies suggest a causal relationship between peripheral and central inflammation as a mechanistic mediator of GWI, other laboratories have reported contrasting findings. For example, Zakirova and colleagues did not observe any significant differences in brain cytokine levels (IL-1β, TNF-α and IFN-γ) in their mouse model of GWI (PB + permethrin) (145). Additionally, plasma levels of these cytokines were reduced, further adding to the uncertainty of whether an inflammatory phenotype is a key feature of GWI rodents (145). Similar findings were reported by our group which saw a reduction in plasma levels of the pro-inflammatory cytokines IL-1α, IL-2, IL-6, IL-12 and IFN-γ in rats that received PB treatment 3 months prior (72). Our follow-up studies also support Zakirova’s findings as we did not observe any difference in hippocampal or PFC cytokine levels in saline-treated rats previously exposed to PB (75). However, we found that a prior history of PB treatment exacerbates the cytokine response in the plasma (TNF-α, cytokine-induced neutrophil chemoattractant 3; CINC-3), hippocampus (IL-1β, IL-12, granulocyte-macrophage colony stimulating factor; GM-CSF) and PFC (IL-12, GM-CSF) when these animals are challenged with a peripheral injection of LPS 3 months after treatment cessation (75). These results suggest that instead of a persistent inflammatory phenotype, GWI may be better characterized as a dysregulation of pro-inflammatory responses and anti-inflammatory feedback mechanisms. Indeed, we also observed that the LPS challenge potentiates ACh efflux in the hippocampus of PB-treated rats 3 months after exposure, which should result in a suppression of pro-inflammatory cytokines, not an exaggeration (75). This potentiation was not specific to an immune challenge, as PB-treated rats also exhibited greater ACh efflux after an immobilization stress challenge relative to vehicle-treated controls, at this delayed timepoint (76). Importantly, these elevations in ACh: 1) were not due to basal differences in ACh levels or alterations in AChE activity within the hippocampus; and 2) only emerged when a physiological stressor was presented months after treatment cessation (75, 76). Such findings mirror the sensitized immune and stress responses observed in GWI patients, further suggesting that dysregulation of the central cholinergic anti-inflammatory pathway is a causative factor in the pathogenesis of GWI. Such results are also consistent with the concept that a latent phenotype is a characteristic feature of GWI. Unfortunately, there are limited studies including such stressors in GWI research, making it difficult to fully elucidate the latent phenotype of GWI and how inflammation may be contributing to the clinical symptoms.

## 5 Lessons from GWI studies in rodents: focus on cognitive studies

Another area of GWI research that could greatly benefit from the inclusion of stressors and thereby reveal a latent phenotype is the behavioral studies aiming to reproduce the cognitive deficits observed in GW Veterans. Though many laboratories have assessed learning and memory in rodents exposed to GW-related chemicals and/or stress, the results from these studies are quite varied; see **Table 1** for outcomes and treatment paradigms. For example, Crawford and coworkers reported that PB + permethrin-treated mice exhibit deficits in the Barnes maze compared to control mice approximately 3 months post treatment (150) and also 15–16 months post treatment (151). However, other behavioral measures yielded more equivocal findings. For example, a study assessing working memory in a rat model of GWI with a delayed alternation task found that stress alone impairs performance, but the combination of PB and stress did not show synergistic effects on working memory (70). Additionally, several groups have utilized novel object recognition (NOR) and novel place recognition testing in GWI models and saw significant impairments in both tasks in a rat model of GWI more than 3 months after treatment cessation (140, 149, 152, 153). Conversely, another group did not observe any deficits in NOR in their rat model of GWI at this 3 month timepoint (141). Interestingly, we observed both results when we assessed NOR performance in PB-treated rats approximately 3 months after treatment cessation, as impairments were only seen in the presence of a physiological stressor (76). Specifically, 3 months after treatment cessation, PB-treated rats that were administered saline (i.p.) before familiar object presentation did not exhibit any deficits in NOR performance 24-hours later (76). However, when these rats were challenged with LPS (i.p.) the following week, PB-treated rats displayed significant impairments in 24-hour memory, while the performance of the vehicle-treated controls was not affected by the LPS challenge (76). Thus, including an immune challenge in our behavioral studies allowed us to unmask hippocampal-dependent memory deficits in our GWI model. It is interesting to speculate that the addition of an immune challenge would have resulted in more consistent behavioral outcomes across these other studies.

**Table 1:** Effects of different GWI treatment paradigms on learning and memory measures in rodents.

GWI paradigm	Testing period(s) relative to Rx	Sex/Species	Outcomes	Reference
PB +/- FS stress	During treatment	Male SD rats	Transient decreases in **alternation performance** in FS group; no individual or additive effects with PB.	(70)
PB +/- PCA for 12 days	Post Rx days 15, 16, 17, 18, 19, 52, 113, 119	Male Wistar rats	Time to escape **water maze** was increased in PB + stress rats at days 16 through 119.	(146)
PB + PER for 10 days	Post Rx days 51, 86, 115	Male CD1 mice	Using the **MWM**, escape latency was reduced in GWI mice during training. In the probe trials, escape latency was reduced in GWI mice at d51 and d 86, but increased at d115.	(147)
PB + PER + DEET + RS for 28 days	Post Rx days 20–28	Male and female C57BL6 mice	In the **MWM**, differences in spatial learning were not observed in GWI Rx mice compared to control mice.	(148)
PB + PER + DEET +/- RRS for 28 days	3 months post Rx	SD rats	GWI Rx alone or in combination with RRS impaired learning during **MWM** training trials and impaired spatial memory in the 24-hour probe trial. In the **NOR**, GWI Rx + RRS rats exhibited reduced exploration times with the novel object but also exhibited decreases in total exploration time; therefore, DI was unaffected.	(141)
PB + PER + DEET + RRS for 28 days	3 months post Rx	SD rats	Compared to control rats, GWI rats exhibited decreased place recognition in the **OLT** and decreased object recognition in the **NOR**. ITI in these tests was one-hour.	(149)
PB + PER for 10 days	Post Rx days 18, 56, 77, 106	Male C57BL6 mice	In the **BM**, long term memory deficits were exclusively observed 106 days post GWI Rx.	(150)
PB + PER for 10 days	15- and 16-months post Rx	Male C57BL6 mice	In the **BM**, path lengths in the training sessions were greater in GWI mice compared to controls. In the 24-probe trial, path lengths were greater in GWI mice 15-months and 16-months post Rx.	(151)
DFP for 5 days	3 months post Rx	Male SD rats	In the **OLT** with a one-hour ITI, DFP mice exhibited reductions in DI compared to control rats.	(152)
DFP for 5 days	6 months post Rx	Male SD rats	In the NOR, DFP mice exhibited decreased DI following a one-hour ITI compared to control rats.	(153)
PB +/-RRS	10 days and 3 months post Rx	Male SD rats	In **CFC**, acquisition of freezing was unchanged but context-induced freezing was reduced in PB + RRS rats 10 days post-Rx. Three-months, post Rx, PB or PB + RRS rats did not exhibit differences in context freezing.	(72)
PB + PER + DEET + RRS for 28 days	3 months post Rx	Male and female B6.Cg-Tg EGFP mice	In the **MWM**, spatial learning and memory was similar in GWI mice and control mice.	(154)
PB +/-RRS	10 days post Rx	Male SD rats	**Cue-conditioned freezing** was reduced in PB-treated rats compared to control rats	(73)
PB + PER + DEET + RRS for 28 days	10 months post Rx	Male SD rats	GWI rats exhibited decreased object recognition in the **NOR** and decreased place recognition in the **OLT**.	(140)
PB +/-RRS	10 days and 3 months post Rx	Male SD rats	**NOR** performance was not affected 10 days post Rx. Three months post Rx, **NOR** performance is impaired in PB + RRS rats 24-hours following LPS administration. In the **MWM**, training performance was unaffected but PB-treated rats exhibited spatial learning deficits in the 24-hour probe trial.	(76)

BM, Barnes maze; CFC, contextual fear conditioning; DEET, N,N,-diethyl-meta-toluamide; DFP, diisopropyl flurophosphate; DI, discrimination index; FS, foot-shock; ITI, inter-trial interval; LPS, lipopolysaccharide; MWM, Morris water maze; NOR, novel object recognition; OLT, object location test; PB, pyridostigmine bromide; PCA, pole-climbing avoidance stress; PER, permethrin; RRS, repeated restraint stress; SD, Sprague-Dawley.

While cognitive performance varied across GWI models and laboratories in the tests described above, more consistent findings have been reported when hippocampal-dependent spatial learning and memory was assessed with water maze tests. For example, while some studies did not identify deficits in the Morris water maze (MWM) in a mouse model of GWI (154), a rat model of GWI exhibited deficits in water maze performance as early as 16 days after treatment cessation and these impairments remained at days 52, 113 and 199 (146). Other studies using a mouse model of GWI reported that MWM performance declines over time, as impairments were not seen at days 20–30 post-treatment (148), but emerged by day 115 (147). While we only assessed MWM performance 3 months after treatment cessation, we observed similar deficits in PB-treated rats, specifically during the 24-hour probe trial (76). Similarly, the group that did not observe deficits in NOR in their rat model of GWI 3 months post exposure reported significant impairments in MWM performance at this timepoint (141). Importantly, relative to the other behavioral paradigms, the MWM is innately stressful due to the swimming component (160), which further supports the idea that stressors must be included in GWI studies in order for cognitive impairments to fully emerge.

**Figure 1:**
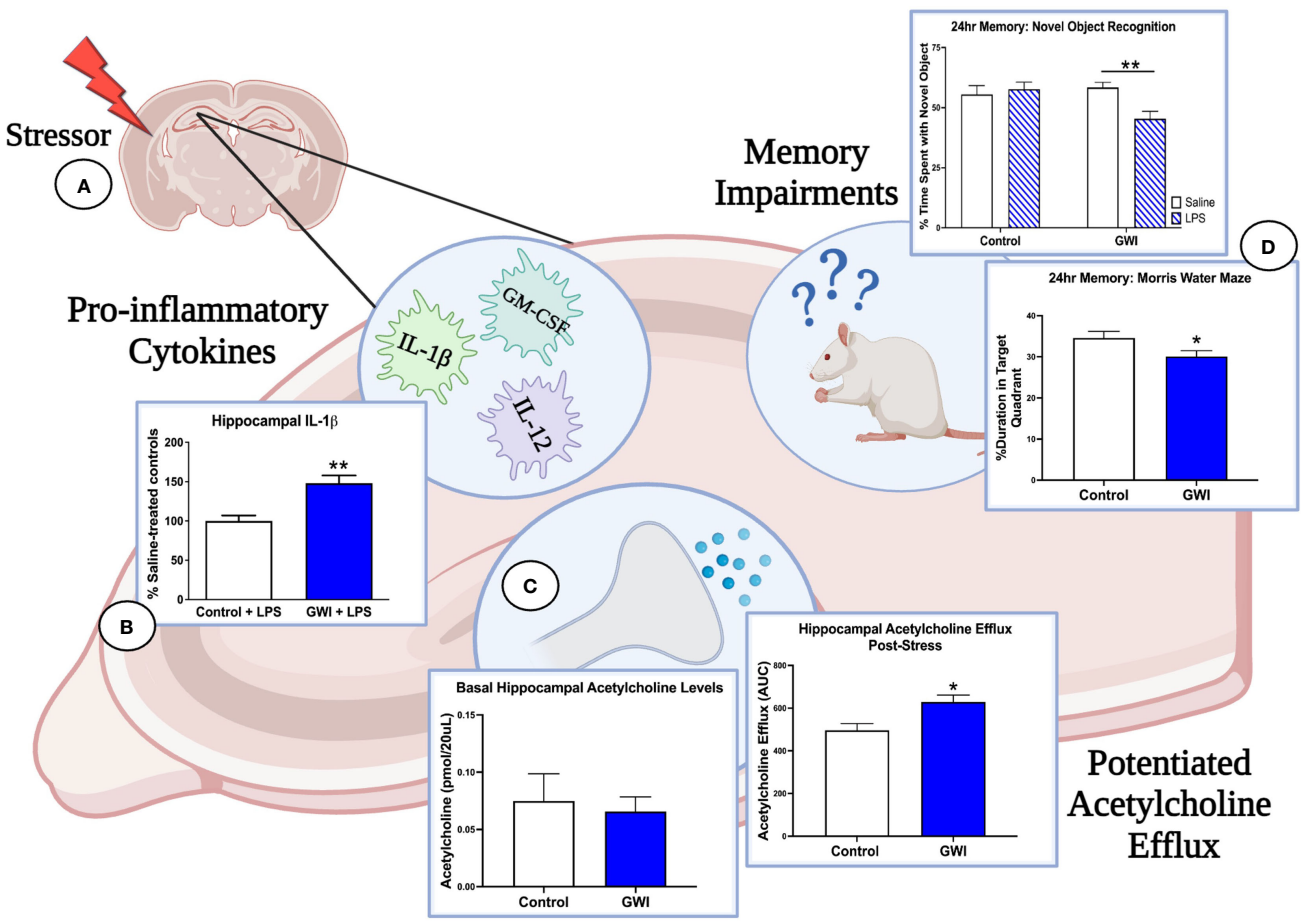
Immune and stress challenges reveal the deleterious effects of GWI on hippocampal function. **(A)** Three months after treatment cessation, our rat model of GWI exhibits sensitized hippocampal responses to both immune and stress challenges, effects that would be missed if our endpoint measures were assessed at resting conditions. **(B)** When challenged with i.p. LPS, rats with a history of PB treatment exhibit exaggerated hippocampal levels of IL-1β (represented in figure), IL-12, and GM-CSF relative to LPS-treated controls, while no differences were observed among saline-treated groups. **(C)** A history of PB treatment also potentiated hippocampal acetylcholine efflux after i.p LPS and a 1-hour immobilization challenge (represented in figure) but did not alter basal levels of acetylcholine in this region. These findings, along with the data shown in Panel A, suggest that PB sensitizes cholinergic responses in the hippocampus but also dysregulates the anti-inflammatory actions of acetylcholine. **(D)** As elevations in pro-inflammatory cytokines and acetylcholine efflux in the hippocampus are known to impair memory consolidation, this dysregulation of the cholinergic anti-inflammatory network are likely driving the 24-hour memory deficits observed in NOR under LPS challenge and MWM performance. Thus, the latent phenotype of GWI and its progressive cognitive impairments will not be fully elucidated until more GWI studies include physiological stressors and unmask these aberrant responses. [Data adapted from 75 and 76].

The stimulus-dependent cognitive impairments that are emerging in GWI rodent models provide further evidence of a latent phenotype of GWI and are consistent with clinical studies in which an exercise challenge is used to exacerbate cognitive deficits in GWI patients (133, 161, 162). Our studies also implicate the cholinergic system as a mechanistic mediator of GWI and suggest that deficits in the central cholinergic anti-inflammatory network may be responsible for the dysregulated immune and stress responses observed in GWI patients (**Figure 1**). In support of this concept, we have reported that hippocampal ACh efflux is potentiated in PB-treated rats when challenged with physiological stressors months later. These enhancements in cholinergic transmission may have cognitive consequences as sustained elevations in ACh levels are known to impair memory consolidation (75, 76, 163). Furthermore, a history of PB treatment appears to dysregulate ACh’s anti-inflammatory actions in the hippocampus as the LPS-induced elevation in ACh efflux is accompanied by greater levels of pro-inflammatory hippocampal cytokines, which also contribute to cognitive deficits (75, 76, 164–166). While we have only assessed the effects of a single physiological stressor in PB-treated rats (i.e., LPS treatment or immobilization stress), GW Veterans continue to face daily life stressors and occasional infections. Thus, the repeated activation of this sensitized cholinergic system within the CNS is likely exacerbated over time and may explain the progressive immune dysregulation, chronic inflammation, and cognitive deficits observed in GWI patients.

The potential sensitization of the central cholinergic system in GWI patients is especially interesting as GW Veterans continue to age. Much like GWI, aging is associated with a “primed” immune system, both in the periphery and CNS, which causes heightened pro-inflammatory responses to stressors (167–169). Additionally, it is well established that aged brains exhibit reduced cholinergic transmission, and the loss of BF cholinergic neurons projecting to brain regions mediating cognition is a hallmark feature of age-related cognitive decline (ARCD) and Alzheimer’s disease (AD) (170). Similar deficits have been observed in GWI studies as 12% of GW Veterans (median age of 48 years) exhibit reduced hippocampal volumes and MCI (58). This rate of MCI is significantly greater than the general population, as the prevalence of MCI in individuals aged 60–64 and 65–69 is 6.7% and 8.4%, respectively (171). These findings suggest that GWI is causing accelerated brain aging, and our rodent studies suggest that this acceleration is due to lasting impairments in the cholinergic anti-inflammatory network. While our rat model of GWI exhibits the same immune sensitivity observed in aging studies, more longitudinal studies are needed to determine if repeated stressors produce similar reductions in hippocampal volume and a loss of cholinergic transmission.

## 6 Potential therapeutics for GWI

Though the mechanisms driving GWI pathology remain undefined, the evidence of immune dysregulation in both preclinical and clinical studies highlight the potential therapeutic benefit of anti-inflammatory interventions for this patient population (**Table 2**). One anti-inflammatory compound that has shown promise in preclinical GWI studies is Lacto-N-fucopentaose III (LNFPIII), an immunomodulatory glycan found in human breast milk (175). Treating a mouse model of GWI with LNFPIII either 7 months or 11 months post-exposure decreased hippocampal levels of IL-6 and increased hippocampal levels of brain-derived neurotrophic factor (BDNF) and nerve growth factor (NGF) (172). Additionally, the same group of investigators found that LNFPIII ameliorates the deficits in hippocampal synaptic plasticity and transmission observed in their mouse model (172, 176). Another family of compounds that have shown anti-inflammatory effects in GWI studies are activators of nuclear factor [erythroid-derived 2]-like 2 (Nrf2), which is a transcription factor that regulates antioxidant responses. This includes melatonin, which normalized Nrf2 levels in a rat model of GWI as well as improved recognition memory (155), and *tert*-butylhydroquinone (tBHQ), which ameliorated reductions in dendritic complexity of dentate granule cells observed in a mouse model of GWI (144). Additionally, various GWI studies have assessed the therapeutic benefit of the antioxidant curcumin. Curcumin has been shown to normalize a number of genes related to mitochondrial respiration in a rat model of GWI, which was associated with reduced hippocampal inflammation, enhanced neurogenesis, and improved performance on cognitive assessments (143). Similar findings were reported in a small clinical study that saw reduced GWI symptom severity in curcumin-treated patients relative to placebo-treated controls (156). Clinical studies have also been performed with the antioxidant L-Carnosine (β-alanine-L-histidine), and GWI patients showed significant improvements in cognitive function after 12 weeks of treatment (157).

**Table 2:** Therapeutic interventions targeting inflammation in GWI.

Intervention	Rationale	Species Tested	Outcomes	Reference
Lacto-N-fucopentaose III	Immunomodulatory glycan with anti-inflammatory properties	Male C57BL6 mice	Reduced hippocampal IL-6 levels. Increased hippocampal BDNF and NGF levels. Ameliorated hippocampal synaptic plasticity.	(172)
Melatonin	Nrf2 activator	Male SD rats	Normalized Nrf2 levels in hippocampus. Reduced astrocyte hypertrophy in hippocampus. Reduced number of activated microglia in hippocampus. Improved performance in NOR and OLT	(155)
*tert*-butylhydroquinone	Nrf2 activator	Male C57BL6 mice	Increases dendritic complexity of dentate gyrate cells.	(144)
Curcumin	Antioxidant	Male SD rats	Normalized genes associated with mitochondrial respiration. Reduced astrocyte hypertrophy in hippocampus. Reduced number of activated microglia in hippocampus. Enhanced hippocampal neurogenesis. Improved performance in OLT and NOR	(143)
		Human	Reduced symptom severity (self-reported).	(156)
L-Carnosine	Antioxidant	Human	Improved cognitive function.	(157)
Low Glutamate Diet	Reduce excitotoxicity by eliminating dietary amino acids	Human	Reduced serum levels of IL-1β.	(173)
			Reduced symptom severity (self-reported).	(174)
Vagal Nerve Stimulation	Stimulate the vagal-mediated cholinergic anti-inflammatory pathway	Male CD1 mice	Reduced GFAP intensity in hippocampus. Improved performance in OLT.	(158)
			Increased hippocampal neurogenesis Moderately improved performance in PST	(159)

BDNF, brain derived neurotrophic factor; GFAP, glial fibrillary acidic protein; IL, interleukin; NGF, nerve growth factor; NOR, novel object recognition; Nrf2, nuclear factor [erythroid-derived 2]-like 2; OLT, object location test; PST, pattern separation task; SD, Sprague Dawley.

Beyond anti-inflammatory compounds, GWI researchers have also assessed the therapeutic potential of vagal nerve stimulation (VNS) as it can increase the vagus nerve’s anti-inflammatory actions. Performing VNS in a mouse model of GWI over 30 weeks after the initial GW chemical exposure reduced the intensity of the astrocytic marker, glial fibrillary acidic protein (GFAP), in the hippocampus and improved cognitive performance in the object location test (158). VNS also ameliorated the reduced hippocampal neurogenesis observed in this GWI model and moderately improved performance in the pattern separation task (159). While this intervention may be promising for the cognitive deficits associated with GWI, VNS may not provide relief for GW Veterans who experience chronic pain as Veterans with GWI that underwent VNS for 10 weeks did not see a reduction in widespread pain or migraine severity or frequency (177). Though some studies have shown positive effects of VNS on disorders associated with chronic pain (178, 179) and migraines (180), other studies suggest that VNS can lower pain thresholds in rodents and humans (181, 182). Thus, future clinical studies are needed to assess the potential benefits of VNS for GWI-related symptoms, especially cognitive impairments, as well as possible adverse effects in this patient population. 

Along with VNS, there are also pharmacological interventions that can restore the cholinergic anti-inflammatory response that may be diminished in GWI patients and potentially alleviate hippocampal neuroinflammation and cognitive impairments. Positive allosteric modulators of α7 nAChRs (α7 PAMs) are emerging as potential therapeutics for a variety of pathologies, ranging from ulcerative colitis to AD, for their anti-inflammatory and pro-cognitive actions (183–185). Unlike agonists that have the potential to elicit receptor desensitization, α7 PAMs are an attractive option as they only augment natural cholinergic signaling (186). Additionally, the allosteric binding site creates higher binding specificity than current pharmacological treatments (187). PNU-120596 is an α7 PAM that has been shown to cross the BBB and inhibit LPS-induced neuroinflammation in rodent studies (188, 189). Furthermore, PNU-120596 has been shown to enhance cognition and prevent the cognitive deficits associated with LPS administration, making it an excellent candidate for future GWI studies (189–193). Collectively, these intervention studies further support the hypothesis that peripheral and CNS inflammation, likely resulting from dysregulation of the cholinergic anti-inflammatory network, is a mechanistic mediator of GWI. More importantly, these studies suggest that restoration of the activity of the cholinergic anti-inflammatory network represents a provocative treatment strategy to ameliorate the progressive cognitive deficits observed in Veterans with GWI.

## 7 Conclusions

In sum, the studies highlighted throughout this review provide strong evidence for a dysregulation of immune and endocrine signaling in GWI, which likely contributes to the progressive cognitive impairments observed in patients. While some studies implicate chronic neuroinflammation as a chief mediator of the cognitive deficits seen in GWI, other studies suggest that such deficits are due to disruptions in central cholinergic signaling that only emerge when a stressor is presented. One major debate within GWI research has been the effect of AChE inhibitors on these cognitive deficits as they should not cross the BBB and affect central cholinergic activity. Our studies support this idea as we do not observe any differences in basal levels of ACh or AChE activity in the hippocampus of our rat model of GWI. However, our studies suggest that PB sensitizes the cholinergic system to physiological stressors, including immune and stress challenges, and these aberrant responses are impairing memory consolidation. Furthermore, the repeated activation of this sensitized cholinergic system over the lifespan likely dysregulates ACh signaling, including its important anti-inflammatory actions, potentiating peripheral and central inflammation and accelerating cognitive decline. These findings are highly consistent with clinical data that report an exacerbation of GWI symptoms, especially cognitive function, when GWI patients are performing an exercise challenge. While this is an exciting area of research, the field needs future studies to include such stressors in preclinical models, as well as alternative stressors in clinical assessments to fully understand the latent phenotype of GWI. These additions will provide better opportunities to determine if peripheral inflammation is driving cognitive deficits or if the central cholinergic system becomes dysregulated through an alternative mechanism. Most importantly, such studies will build upon this strong foundation of GWI research and help identify potential therapeutics that will slow or stop this debilitating disease.

## Author contributions

HB: Conceptualization, Formal analysis, Funding acquisition, Writing – original draft, Writing – review & editing. LR: Conceptualization, Formal analysis, Funding acquisition, Writing – original draft, Writing – review & editing.
